# Reversible postoperative vision loss (POVL): A tale of four patients

**DOI:** 10.5339/qmj.2024.37

**Published:** 2024-10-07

**Authors:** Nissar Shaikh, Seema Nahid, Firdos Ummunnisa, Umm E Amara, Umme Nasrah, Azha Fatima, Fateen Shareef, Abul Rahman Balal

**Affiliations:** 1 Hamad Medical Corporation, Doha, Qatar *Email: snahid@hamad.qa; 2 Halima AL Tamim OBGY Clinic, Doha, Qatar; 3 Apollo Institute of Medical Sciences, Hyderabad, India; 4 Deccan Medical College, Hyderabad, India; 5 Kamineni Institute of Medical Sciences, Hyderabad, India

**Keywords:** Hypertension, hypotension, magnetic resonance imaging, postoperative vision loss, reversible

## Abstract

**Background:**

Posterior reversible encephalopathy syndrome (PRES) is a clinic-imaging entity. PRES is rarely reported in the perioperative period to cause reversible postoperative vision loss (POVL). It is reported in the literature in the form of case reports for spinal and cardiac surgeries and eclampsia patients. The suggested diagnostic criteria for PRES are: (i) acute onset of neurological symptoms and signs; (ii) specific findings of vasogenic cerebral edema upon imaging studies; and (iii) reversibility of signs and symptoms as well as image study findings. We report a case series of four patients undergoing other than spinal, cardiac, or orthopedic surgeries who developed PRES and had reversible POVL.

**Cases:**

The first case was a young female who had laparoscopic sleeve gastrectomy, had extreme hypertension at induction of anesthesia, had surgery and developed POVL after a few hours in the postoperative period, and had convulsions diagnosed to have PRES after computed tomography (CT) and magnetic resonance imaging (MRI). Managed with blood pressure and seizure control, vision returned gradually from 2nd postoperative day. The second case was also a young female who had appendicitis, requiring an appendectomy. Complicated by septic shock. Post-surgery, she was extubated after 1 day and immediately complained of total blindness. Local causes were ruled out, and an MRI diagnosed PRES. With supportive therapy, her vision started to return by Day 3 with improved normal vision. The third case was a female with recently diagnosed diabetes mellitus who presented with right upper limb embolic ischemia and had an embolectomy with a return of circulation. Her blood pressure was high and reached up to 200 mmHg after induction of anesthesia, which was controlled with deep anesthesia and a labetalol infusion in the perioperative period. After 8 h in the postoperative period, she was awake but searching for available objects. Relatives complained that she was unable to see. Local and fundus examinations were normal. She was awake but blind. Imaging studies confirmed PRES. Blood pressure was controlled using a labetalol infusion and continued supportive therapy. By Day 3, her vision became normal. The fourth case was an elderly patient who had hypertension, type 2 diabetes mellitus, and coronary artery disease. He underwent a right carotid endarterectomy under general anesthesia. He had severe hypertension in the perioperative area and blood pressure was controlled using a labetalol infusion. The surgery went smoothly. After 3 h, he had a loss of vision. Imaging studies confirmed PRES. His blood pressure was kept normal. After 2 days, his vision gradually returned to normal. Follow-up MRIs in the outpatient clinic for all four patients normalized in due time.

**Conclusion:**

Extremes of hypertension and/or hypotension in the perioperative period can cause PRES, which may lead to reversible POVL.

## Introduction

Post-operative vision loss (POVL) after non-ocular surgeries is a serious and worrisome complication not only for the patient but for the treating team. POVL can frequently occur due to various etiologies ranging from corneal abrasion to the posterior reversible encephalopathy syndrome (PRES).^1^ The reported incidence of POVL ranges from 0.5 to 1%. Cardiac and spinal surgeries are the riskiest surgeries for POVL.^2^ In the majority of patients, POVL is due to local ophthalmological complications such as anterior and posterior ischemic retinopathy due to the positioning, which causes permanent loss of vision. POVL due to posterior reversible encephalopathy may cause transient and reversible loss of vision in most of the patients.^1,2^ PRES is reported to cause reversible POVL in spinal surgeries and eclampsia patients in the form of case reports.^1^ PRES was first described by Hinchey in 1996; it is defined by the presence of a clinical neurological disorder and specific imaging findings.^3,4^ Fisher and Schmutzhard suggested the diagnostic criteria for PRES as (i) acute onset of neurological symptoms and signs; (ii) specific findings of vasogenic cerebral edema upon imaging studies; and (iii) reversibility of signs and symptoms as well as image study findings.^5^ PRES is more common in females; it affects all ages and is more frequent in young patients. Most frequently, patients with PRES will have encephalopathy in up to 92%, altered consciousness in up to 90%, acute hypertension and/or extreme fluctuations in blood pressure in up to 80%, siezures in up to 74%, acute vision loss and/or visual disturbances in up to 67%, and headache in up to 53%.^5^ Imaging studies, particularly magnetic resonance imaging (MRI), show bilateral symmetrical white matter abnormalities in vascular watershed areas in posterior cerebral hemispheres, frequently affecting occipital-parietal lobes, in association with widespread or localized vasogenic edema. In a typical case, the changes are not limited to the posterior cerebrocortex^4^; they can involve asymmetrical isolated frontal lobes with cortical lesions. Vasogenic cerebral edema is not necessarily localized to the posterior cerebrum white matter and can appear in watershed zones other than parieto-occipital regions, the thalamus, and sometimes in the anterior circulation.^6^ We report a series of four patients with reversible POVL after non-ophthalmic surgery.

### Case 1

**Figure 1.**
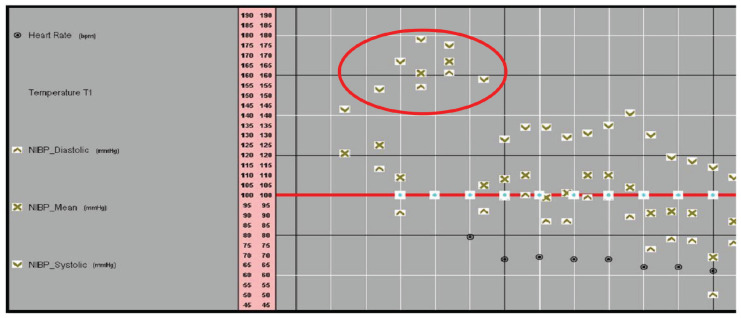
Showing hypertension at induction of anesthesia.

A 42-year-old female was admitted electively for sleeve gastrectomy. She was moderately obese (body mass index [BMI] 35.3 kg/m^2^), had poorly controlled hypertension on four antihypertensive medications, had diabetes mellitus type 2 on oral hypoglycemic medications, and had hypothyroidism on replacement therapy. Anesthesia was induced after connecting to standard monitoring, using fentanyl, lidocaine, and propofol, and intubated under the effect of rocuronium. Pre-intubation blood pressure was 150/110. Immediately after induction for 18 min, the blood pressure reached a systolic of 200 mmHg (Figure 1), which was controlled using deeper anesthesia. She was extubated at the end of the surgery and transferred to the surgical intensive care unit.

Postoperatively, she was awake and had stable hemodynamics. After 2 h, she was complaining of total blindness, but was conscious and moving all her limbs, and the ophthalmological examination of the cornea, anterior, and posterior chambers was normal. The echocardiogram was normal and did not show any emboli. She was transferred to a computed tomography (CT) scan, and while on the CT table, she developed a generalized tonic colonic seizure that aborted spontaneously. The CT brain showed features of PRES and was confirmed by MRI. The CT brain angiography and MRI ruled out vascular abnormalities such as vasculitis. Laboratory work, including the erythrocyte sedimentation rate (ESR), C-reactive protein (CRP), and anti-neutrophil cytoplasmic antibody (ANCA) test, were negative. She was started on an anticonvulsant (Levetiracetam) and maintained a systolic blood pressure of 140–160 mmHg. She was extubated after 24 h; the patient was awake but unable to see anything. Her vasculitis workup was negative. From the 2nd day’s evening hours, she was able to see moving objects, and her vision gradually improved. By Day 4, her vision became normal. She was transferred to the ward and subsequently discharged home by Day 7. Patient’s follow-up in outpatient at 1, 3, 6, 9, and 12 months was normal, and imaging showed reversed to normal.

### Case 2

**Figure 2.**
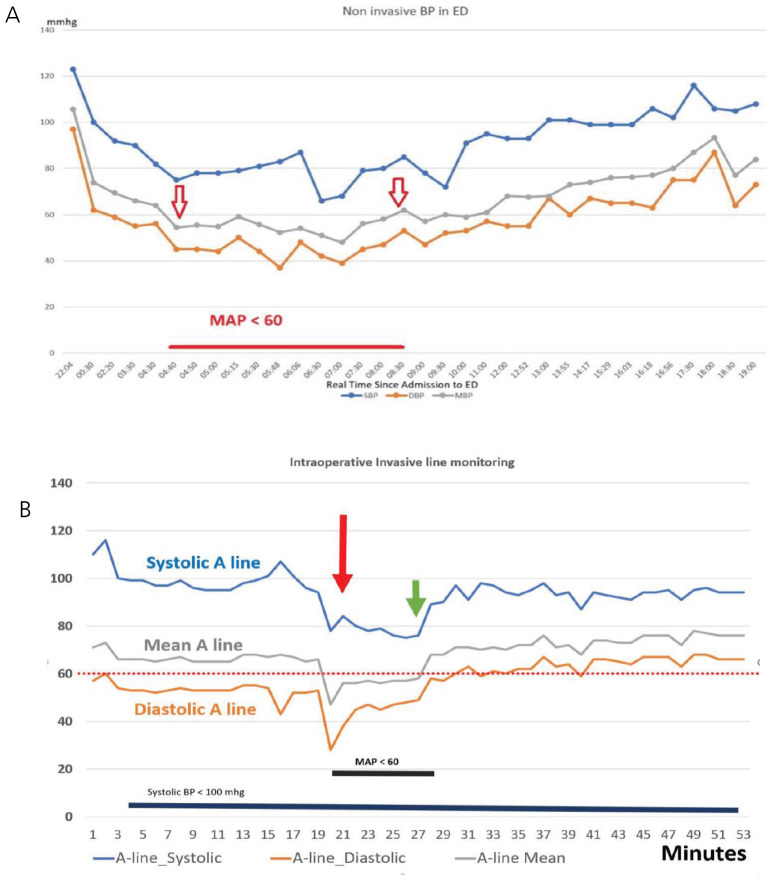
(A) Showing hypotension in the Emergency Department. (B) Showing intraoperative hypotension.

A 36-year-old female without any comorbidities presented to the emergency department (ED) with abdominal pain, subsequently diagnosed as acute appendicitis. She had bouts of hypotension in the ED related to dehydration (Figures 2A, B) and was treated with fluid boluses and ephedrine.

She was taken for laparoscopic appendectomy, and after pneumatic inflation of the abdomen, she had hypotension for 5 min, requiring a noradrenaline infusion. She was admitted post-procedure to the surgical intensive care unit, where she was intubated, ventilated, and maintained on a noradrenaline infusion. She was weaned from the ventilator and vasopressor by the next day and extubated. Immediately after extubation, the patient complained of “not being able to see anything.” On the local examination, pupils were reactive to light, and the fundus exam was normal. The echocardiogram was normal. She remained awake, moving all her limbs, but totally blind. CT of the brain was normal, and CT angiogram and MRI did not show any vasculitis finding or vascular abnormality, but MRI showed bilateral occipital hypodensities consistent with PRES. Her vasculitis (ANCA was negative) and thrombophilia markers (anti-phospholipid antibodies were negative, protein C and S within the normal range) were negative. On Day 3, she started to appreciate hand movements and perception of light; her vision gradually improved to normal by Day 5. She was transferred from the surgical intensive care unit to the ward and then discharged home. Follow-up after 3, 6, 9 months, and 1 year, she remained healthy, and the MRI became normal.

### Case 3

**Figure 3.**
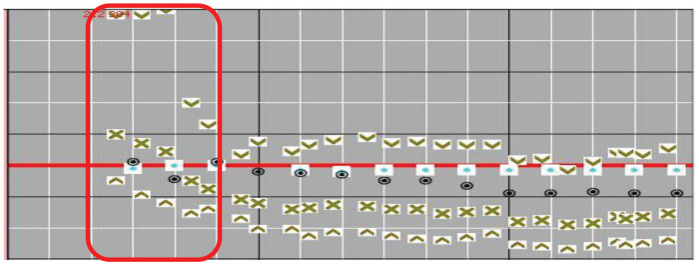
Showing hypertension at induction.

A 45-year-old female newly diagnosed with type 2 diabetes mellitus, presented to the ED with right upper limb pain for 1 day associated with hand numbness and coldness. She was hypertensive, with a blood pressure of 170/116 controlled on labetalol boluses. A CT angiogram of the upper limb showed obliteration of the right distal brachial, radial, and ulnar arteries. She immediately underwent embolectomy and patch angioplasty of the brachial artery. After induction, her blood pressure readings were up to systolic 200 mmHg (Figure 3). Postoperatively, she was admitted to the surgical intensive care unit. After admission, she remained hypertensive, requiring a labetalol infusion. She was started on therapeutic anticoagulation with a heparin infusion, and pain was controlled using patient-controlled analgesia. She was awake and moving all her limbs. After 8 h, relatives complained that the patient is blind and not able to identify objects. On examination, she was blind and had no local signs of inflammation or trauma. The fundus examination was normal. The echocardiogram was normal and did not show any thrombus or emboli.

The CT brain showed bilateral occipital hypodensities, and an MRI confirmed PRES. The CT angiogram and MRI showed normal cerebral vessels and no features of vasculitis. Her systolic blood pressure was kept between 140 and 160 mmHg using a labetalol infusion, and adequate analgesia was maintained with IV boluses of fentanyl. Thrombophilia and vasculitis markers (ESR, CRP, and ANCA) were negative. Her vision started to improve by Day 3 and completely returned to normal by Day 5. She was transferred to the ward and subsequently discharged. The follow-up after 1 year showed no neurological or vision abnormalities, and the MRI was normal.

### Case 4

**Figure 4.**
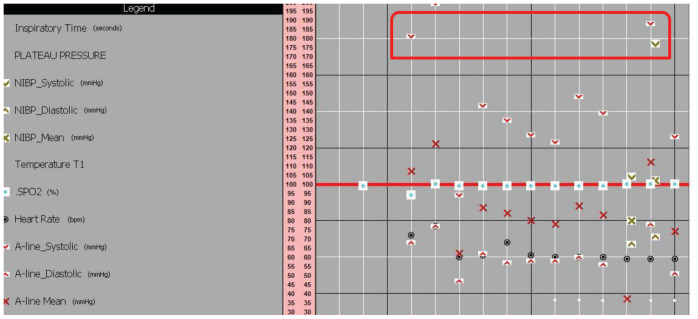
Hypertension at induction of anesthesia.

A 68-year-old male known to have hypertension, type 2 diabetes mellitus, coronary artery disease post coronary artery bypass graft, and carotid artery stenosis was admitted to the surgical intensive care unit postoperatively after right carotid endarterectomy and angioplasty. He had hypertension after induction of anesthesia for 10 min, which was controlled using labetalol and deepening of anesthesia (Figure 4).

Postoperatively, the patient was admitted to the surgical intensive care unit. He was awake and hemodynamically stable. After 3 h of Surgical Intensive Care Unit (SICU) admission, he was searching for objects and complained of vision loss. On examination, he was awake and following commands, with no abnormalities in the cornea or any signs of inflammation. The fundus examination of both eyes was within normal limits. The echocardiogram was normal. CT and CT angiography of the brain were normal, and MRI showed features of PRES and vascular abnormalities. He was continued on supportive care. He was hypotensive and not responding to intravenous fluids; therefore, he was started on a noradrenaline infusion. The echocardiogram did not show any emboli or new wall motion abnormalities, and the vasculitis markers (ESR, CRP, and ANCA) were negative and normal. On Day 2, the patient started to appreciate moving objects. By Day 3, he was able to see the wall clock, and his vision returned to normal by Day 5. He was discharged home and followed up in the outpatient clinic at 3, 6, 9 months, and 1 year; he did not show any abnormalities. The MRI was normal at 6 months.

## Discussion

POVL has a devastating effect on the clinical care team in addition to the patient and family. The incidence of POVL ranges from 0.056 to 1.3%, more frequently after cardiac and spinal surgeries, and frequently occurs due to ischemic retinopathy.^1^ Overall risk factors for POVL are male gender, prone position during surgery, prolonged duration of surgery and unconsciousness, anemia, blood transfusion, and the decreased use of colloids.^7^ A few modifiable factors for POVL are obesity, coronary artery disease, diabetes mellitus type 2, hyperlipidemia, and smoking.^2,7^ Cortical blindness, also called Anton’s syndrome or PRES, causes POVL. POVL is often reversible, while permanent vision loss has been observed in PRES patients with eclampsia. POVL resulting from PRES during the postoperative period is infrequently documented and is limited to case reports. It is reported in the literature in the form of case reports, and up to date, only 12 cases have been found in the literature. Search criteria include the keywords postoperative blindness and PRES in Google Scholar and PubMed Search.^8^ According to the largest US study on POVL, PRES blindness affects 0.0038% of POVL cases.^9^ They also reported ages less than 18 years, a higher Charlson index, elective admission, and spinal and cardiac surgeries were the risk factors for cortical blindness.^9^

The etiological pathophysiology of postoperative blindness is examined in order to differentiate and better understand it. The corneal ulcer can cause blindness due to decreased tear secretion or decreased corneal protection. Corneal ulcerations are painful. This ulceration can be unilateral or bilateral. The risk of a corneal ulcer is increased in lateral or prone positions, longer duration of surgery. It is easy to diagnose by local ophthalmic examination. Ischemic optic neuropathy (ION) is the most frequent cause of permanent postoperative blindness. ION can be anterior or posterior, depending on the involvement of the optic nerve. The primary risk factors for ION are older patients, transfusions, and the Trendelenburg and prone positions. A fundus examination can diagnose ION, which is often unilateral. Anterior ION fundus examinations will reveal optic disc edema and peripapillary hemorrhages, while posterior ION fundus examinations will reveal pale optic nerves. It will not have any pupillary reflexes. ION is a slow, painless loss of eyesight.^10^

Central retinal artery occlusion (CRAO) causes postoperative blindness in a relatively small percentage, commonly associated with embolic phenomena, and it is a unilateral and painful loss of vision. Elderly males, hypertension, diabetes mellitus, coronary artery disease, hyperlipidemia, and smoking are risk factors for CRAO. The ophthalmic fundus examination used to diagnose unilateral CRAO will reveal characteristic cherry-red patches on the macula associated with ischemic retina.^11^ Vasculitis is a multisystem disease; apart from the eyes it also affects other organs in the body. The patient may present with other systemic complaints. The biomarkers ESR and CRP will be raised, but they are not specific, whereas ANCA is specific and useful in ruling out vasculitis. The imaging studies of the brain will reveal vascular abnormalities with complications such as hemorrhage or infarctions, at the same time, the local ophthalmic fundus examination will show vasculitis changes in retina.^12^

PRES causing blindness is rare. Patients will have bilateral, painless vision loss within a few hours in the postoperative period. The painless blindness is frequently bilateral. Commonly, there will be a history of severe hypertension and rarely hypotension in the perioperative period. Other risk factors described for the occurrence of PRES and blindness include a longer duration of anesthesia and surgical position. Ophthalmic fundus examinations will be normal, rarely if blood pressure is not controlled, it can show edema. The echocardiogram may show chronic hypertensive changes without any thrombus or emboli. The imaging studies, particularly the MRI brain, are more sensitive in detecting the symmetrical occipital lesions in PRES compared to the CT scan.^13^ The brain’s vascular structure will be normal. These brain lesions in PRES patients frequently normalize in a few months.^13^

All of our patients who underwent non-cardiac or spinal procedures were older than eighteen, and the anesthesia was not prolonged. Even though two of our cases involved the Trendelenburg position, the procedure did not take longer than standard procedure time. The fundus and eye examinations were normal. The loss of vision was bilateral and painless. The echocardiogram in all our patients was in the normal range and did not show any emboli or thrombus. The MRI brains of all four patients showed typical findings of PRES.

**Figure 5.**
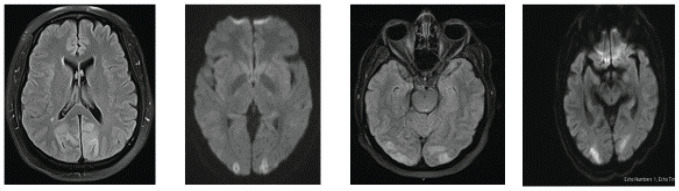
MRI brain of 1 to 4th case from left to right showing PRES.

PRES occurs due to edema and hypoperfusion in the occipital lobe, with bilateral peculiar findings in the imaging studies, particularly the MRI of the brain. CT brain imaging may be normal in PRES patients.^14^ All our patients had typical radiological findings in the bilateral occipital region (Figure 5), which were completely reversed after a few months, and vision returned normally within a few days of occurrence. Most of our patients (3) had significant hypertension in the perioperative period; one patient had sepsis and hypotension.

PRES is increasingly recognized with the recent availability and improvement in image sciences and its awareness.^15,16^ In the majority of case reports of POVL due to PRES in the perioperative period, patients had severe hypertension.^17^ Gupta et al. reported a case of PRES-causing POVL due to hypotension with full recovery.^18^ Visual disturbances are reported in up to 33% of all PRES-reported patients with different etiologies and generally good prognoses. There was normalization of vision in all reported cases and in our patients within days of POVL, which will be explained by the resolution of edema in the posterior lobe or cortical fibers in the visual pathway with supportive care, control of hypertension, and anticonvulsant therapy.^19^

Our institution is the only adult tertiary health care facility in Doha, Qatar, and it is a high-volume center for the described cases. In the author’s knowledge over decades, this type of reversible postoperative blindness has not occurred, and the literature is also lacking about any such cases reported from the country.

Further multidisciplinary, multicenter larger comparative studies are needed to know the epidemiology risk factors and management of PRES blindness in the postoperative period.

## Conclusion

PRES can cause reversible POVL in other than cardiac, spine, and orthopedic surgeries. Blood pressure drops and fall is a risk factor for PRES and postoperative blindness. Standard of care should be followed during the preoperative and intraoperative periods and avoiding extreme changes in blood pressure—either hypertension or hypotension—is essential in the perioperative period for preventing PRES and occurrence of POVL.

## Conflict of Interest Statement

Not applicable.

## Declaration

Medical research center of our institute has given permission (MRC-04-23-520) for publication of case series.

## Authors’ Contributions

NS and SN wrote the Abstract and Background. AB prepared figures and wrote the discussion part. FU, UA, UN, AF, and FS reviewed the manuscript.

## Ethics Approval and Consent to Participate

Done.

## Availability of Data and Materials

Present.
